# The Impact of “The Magic Glasses Opisthorchiasis” on Schoolchildren’s Knowledge, Attitudes and Practices Surrounding *Opisthorchis viverrini* in the Lower Mekong Basin, a Cluster-Randomised Controlled Trial

**DOI:** 10.3390/tropicalmed11070174

**Published:** 2026-06-24

**Authors:** Suji Y. O’Connor, Mary Lorraine Mationg, Matthew J. Kelly, Gail M. Williams, Archie C. A. Clements, Banchob Sripa, Somphou Sayasone, Virak Khieu, Kinley Wangdi, Donald E. Stewart, Sirikachorn Tangkawattana, Apiporn T. Suwannatrai, Vanthanom Savathdy, Visal Khieu, Peter Odermatt, Catherine A. Gordon, Sangduan Wannachart, Donald P. McManus, Darren J. Gray

**Affiliations:** 1 National Centre for Epidemiology and Population Health, Australian National University, Canberra, ACT 2601, Australia; 2 School of Clinical Medicine, University of New South Wales, Sydney, NSW 2033, Australia; 3 Population Health Program, QIMR Berghofer, Brisbane, QLD 4006, Australia; 4 School of Public Health, University of Queensland, Brisbane, QLD 4006, Australia; 5 School of Biological Sciences, Queen’s University Belfast, Belfast BT7 1NN, UK; 6 Department of Tropical Medicine, Faculty of Medicine, Khon Kaen University, Khon Kaen 40002, Thailand; 7 Tropical Disease Research Center, Faculty of Medicine, Khon Kaen University, Khon Kaen 40002, Thailand; 8 Lao Tropical and Public Health Institute, Ministry of Health, Vientiane 01000, Laos; 9 National Center for Parasitology, Entomology and Malaria Control, Ministry of Health, Phnom Penh 12101, Cambodia; 10 HEAL Global Research Centre, Health Research Institute, Faculty of Health, University of Canberra, Canberra, ACT 2617, Australia; 11 School of Medicine and Dentistry, Griffith University, Gold Coast, QLD 4215, Australia; 12 Faculty of Veterinary Medicine, Khon Kaen University, Khon Kaen 40002, Thailand; 13 Department of Parasitology, Faculty of Medicine, Khon Kaen University, Khon Kaen 40002, Thailand; 14 Swiss Tropical and Public Health Institute, 4123 Allschwil, Switzerland; 15 University of Basel, 4001 Basel, Switzerland; 16 School of Medicine, University of Queensland, Brisbane, QLD 4072, Australia; 17 School of Biological Sciences, Queensland University of Technology, Brisbane, QLD 4001, Australia; 18 Centre for Tropical Health and Emerging Diseases, QIMR Berghofer, Brisbane, QLD 4006, Australia

**Keywords:** *Opisthorchis viverrini*, opisthorchiasis, randomised controlled trial, school-based health education, Magic Glasses, health promotion, neglected tropical diseases

## Abstract

*Opisthorchis viverrini* (OV) is a liver fluke endemic to the Lower Mekong Basin. Infections often begin in childhood and are causally linked to cholangiocarcinoma, an often-fatal bile duct cancer. Anthelmintic treatment is the primary control strategy, but infection can recur. Therefore, additional strategies are needed. This study assessed the impact of “The Magic Glasses Opisthorchiasis” (MGO), a cartoon-based intervention, on schoolchildren’s OV-related knowledge, attitudes and practices (KAP). A cluster (school)-randomised controlled trial was conducted in Cambodia, Laos and Thailand. Clusters were randomised into either school health education only or with MGO. OV KAP was measured using a standardised questionnaire. FGDs and interviews were also conducted in intervention schools with schoolchildren, parents, and teachers. Cambodia intervention knowledge and attitude scores improved by 19.2 (*p* < 0.001) and 25.3 (*p* < 0.001) percentage points, respectively, relative to the control. Laos intervention knowledge and attitude scores improved by 19.0 (*p* < 0.001) and 14.2 (*p* < 0.001) percentage points. However, Thailand’s intervention knowledge and attitude scores declined by 23.3 (*p* < 0.001) and 15.8 percentage points (*p* < 0.001). There were no improvements in behaviour scores in any country, but parents and schoolchildren in Cambodia and Laos reported improved fish preparation practices, suggesting positive spillover effects from MGO. The findings support MGO as an effective tool for school-based health education.

## 1. Introduction

*Opisthorchis viverrini* (OV) is a foodborne trematode estimated to affect over 10 million people in Southeast Asia [1]. The majority of cases occur in the Lower Mekong Basin [1], a region which includes Cambodia, the Lao People’s Democratic Republic (henceforth Laos), Northeast Thailand and Southern Vietnam. OV prevalence is consistently high in the LMB [2,3,4], reaching almost 90% in some provinces [5]. A 2018 study reported a prevalence of 75.8–80.2% in children aged 5–10 years old in Khammouane Province, Laos [6]. OV transmission occurs via the consumption of raw or undercooked infected freshwater cyprinid fish [7], which are common in local dishes such as *koi pla* and *padaek plara*. Acute and mild OV infections can cause fever, fatigue, diarrhoea and constipation, but they are often asymptomatic, evading detection [8]. Untreated, infections can persist for decades [9]. High-intensity or chronic infections can cause hepatobiliary abnormalities causally linked to cholangiocarcinoma, an often-fatal bile duct cancer [9,10]. A recent study (2024) reported that the likelihood of suspected CCA was threefold in OV-positive participants, compared to uninfected participants [5].

The primary OV control strategy is anthelmintic treatment with praziquantel, which has 90% efficacy [11,12]. However, hepatobiliary abnormalities linked to chronic or repeated OV infections can persist after treatment [13], and reinfection can occur [14]. Therefore, additional control strategies addressing the primary dietary risk factor are needed. As OV infection often begins in childhood [6,15,16], early-age interventions are particularly important.

The “Magic Glasses” (MG) is a school-based health education package originally developed to improve children’s knowledge, attitudes and practices (KAP) regarding soil-transmitted helminths (STHs) in China and reduce infections [17]. The primary intervention component is an educational cartoon set in a local context with student protagonists who learn important STH health messages and help their community. Additional components of the intervention package include a comic pamphlet with key messages from the cartoon and reinforcement activities [17]. A 2010–2011 cluster-randomised controlled trial (cluster-RCT) in Hunan Province, China, found that MG increased children’s mean STH KAP scores by 24.9 percentage points and achieved a 50% reduction in STH incidence [17]. The cartoon was adapted for the Philippines in 2015 and achieved significant improvements in knowledge and behaviour scores in intervention schools (increases of 5.3 and 1.1 percentage points, respectively, *p* < 0.01) and a 60% reduction in STH incidence in schools with a baseline prevalence of ≤15% [18]. Recognising the success of previous MGs, a new cartoon, “The Magic Glasses Opisthorchiasis” (henceforth MGO), was developed for OV in the LMB. Unlike previous MG approaches, MGO was designed to target multiple countries—Cambodia, Laos and Thailand—that have high OV burden and similar environments [19,20]. The storyline was adapted to focus on OV health messages, informed by literature reviews and local expertise. The cartoon design was set in the Lower Mekong environment and included cultural elements such as attire and culinary dishes, informed by local stakeholders. This study aimed to evaluate the impact of MGO on schoolchildren’s OV-related knowledge, attitudes and practices (KAP) in Cambodia, Laos and Thailand. The outcomes of this study will inform the generalisability of a new MG video for implementation in multiple countries and the adaptability of the intervention to other infectious diseases.

## 2. Methods

The full description of the study procedures can be accessed in the published study protocol in Supplementary Material S1 [14].

### 2.1. Intervention

MGO targeted OV KAP among primary schoolchildren in Cambodia, Laos and Thailand. Health messages were informed by literature reviews on risk factors and OV KAP in the LMB. The cartoon storyboard and script were reviewed by stakeholders from all study countries for cultural appropriateness and health messaging, and feedback was incorporated into the final version. The cartoon was first developed for Thailand and then adapted for Cambodia and Laos. The script, protagonists and storyline were identical across versions, but cultural aspects, such as national flags and characters’ attire, were changed to make the cartoon relatable for each study setting. Dialogue was recorded in the local language for each country.

### 2.2. Study Design

Cluster-RCTs targeting primary schoolchildren were conducted in Preah Vihear Province, Cambodia; Savannakhet Province, Laos; and Khon Kaen Province, Thailand.

This study was designed with 80% power to detect an intervention effect of five percentage points in each trial, assuming a baseline OV knowledge score of 30%. Accounting for cluster sampling, sample size calculations assumed a design effect of 2 and 10% attrition. The target sample size was 228 schoolchildren per country. In line with previous studies, schoolchildren in Grades 3–6 (aged 8–12 years) were eligible for this study [17,18]. Two schools (clusters) were recruited in Cambodia, six in Laos and six in Thailand. Schools were randomly allocated to control or intervention arms. Due to the nature of this study, blinding was not possible. SYO performed random allocation using the Microsoft Excel (Microsoft 365, Redmond, Washington, USA) RAND function. Schools within 3 kilometres of each other were allocated as a single cluster to minimise contamination between control and intervention arms. Intervention arms received MGO in addition to existing school-based water, sanitation and hygiene (WASH) education. Control arms received only existing school WASH education.

This study was conducted between 2023 and 2024. Study timings varied by country (Table 1). Follow-up was conducted 9 months after baseline in Cambodia and Thailand and 13 months after baseline in Laos due to logistical delays.

### 2.3. Measures

Intervention and control participants undertook a self-reported KAP questionnaire on OV at baseline. The questionnaire was administered by trained field staff. The questionnaire was adapted from a validated version used for STH KAP assessment in previous MG studies. Questions from the original STH questionnaire that related to disease-specific risk factors, transmission, symptoms, prevention and treatment were changed to include OV health information. The adapted questionnaire was then reviewed by disease experts to ensure accuracy. Following the completion of the baseline questionnaire, MGO was administered in the intervention arms (Table 2). A reinforcement MGO session was held 6–8 weeks after initial delivery.

At follow-up, the OV KAP questionnaire was re-administered in the intervention and control arms, and qualitative data were collected in the intervention arms. Trained field staff facilitated focus group discussions (FGDs) with students and key informant interviews (KIIs) with teachers using a question guide (see Table 3) to explore the direct impacts of MGO on OV KAP. FGDs were also held with parents to explore knowledge transfer.

Schools in the control arm received the intervention after the follow-up OV KAP questionnaire was complete.

The primary outcome measurement was the impact of MGO on students’ self-reported OV KAP. Secondary outcomes were the impact of MGO on teachers’ and parents’ OV KAP.

### 2.4. Ethics Statement

This study was approved by the Australian National University Human Ethics Committee (approval number 2022/507), the Cambodia Ministry of Health National Ethics Committee for Health Research (approval number 2022/507), the Laos Ministry of Health National Ethics Committee for Health Research (approval number 2021.54), and Khon Kaen University Ethics Committee for Human Research (approval number 4.2.03:16/2565). This study was registered at the Australian New Zealand Clinical Trials Registry on 13 March 2023: ACTRN12623000271606 (www.anzctr.org.au). Permission to conduct this study was obtained from provincial and school officials. Consent and assent were obtained prior to participant enrolment. Staff training workshops were held in each country prior to study commencement to ensure that standard operating procedures were maintained.

### 2.5. Data Analyses

#### 2.5.1. Quantitative Analysis

Completed questionnaires were double-entered into an encrypted RedCap electronic database (version 14.9.5) hosted by the University of Queensland. Original paper questionnaires in Cambodia, Laos and Thailand were stored in secured facilities in the Cambodian Ministry of Health, the Lao Ministry of Health, and Khon Kaen University, respectively.

Descriptive analyses assessed participant age and sex distribution. A scoring chart was developed for each component of the questionnaire. In the knowledge and attitude components, scores were awarded for correct responses, with maximum possible scores of 15 and 7, respectively. Higher scores indicate higher levels of knowledge and attitude. In the behaviour component, scores were awarded for risk-taking behaviour, with a maximum possible score of 8. Higher scores indicate less safe behaviour. For specific scores and weighting, see Supplementary Material S2. Data was analysed by country. Scores were converted to percentage points for analyses within and between study arms. Data were analysed in STATA 18 (StataCorp LLC., College Station, TX, USA) using generalised estimating equation (GEE) regression models with 95% confidence intervals (CIs) to assess the main and interaction effects of study arm and timepoint on KAP questionnaire responses, accounting for school clustering and repeated measures on the same individuals. GEE analyses specified a Gaussian model with an identity link function to allow for the direct interpretations of scores.

#### 2.5.2. Qualitative Analysis

All FGDs and KIIs were transcribed in the local language and then translated into English. Translated data were extracted into nVivo 14 (QSR International, Denver, CO, USA). Qualitative analyses were performed for each country using the six-step method outlined by Braun and Clarke [21]. Data were examined and coded into broad categories using an inductive approach guided by the research aims; coding categories were searched for common themes, and these were reviewed and examined in terms of importance. Qualitative results are presented for each country according to the themes that emerged in each setting.

## 3. Results

### 3.1. Participants

#### 3.1.1. Quantitative

**Figure 1:**
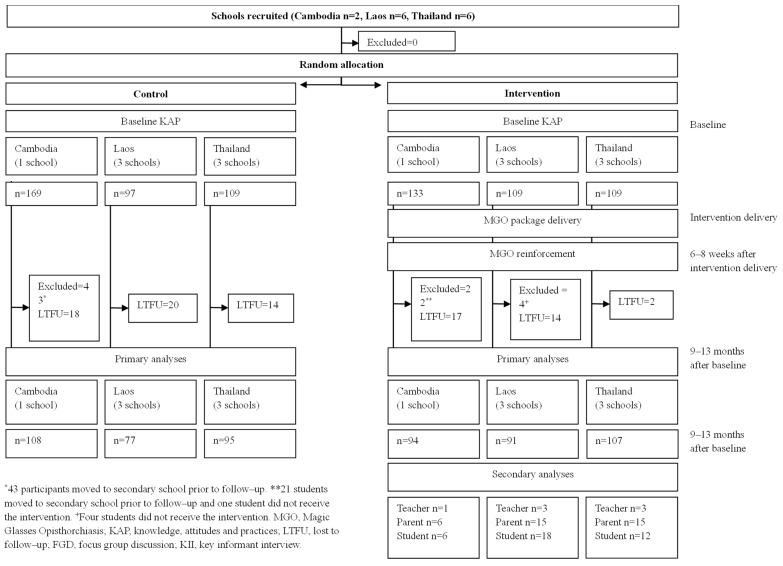
Trial profile.

A total of 726 schoolchildren were recruited into this study (see Figure 1). In Cambodia, one student was excluded because they did not receive the intervention, and 64 students were excluded as they moved to secondary school during the study period; 35 were lost to follow-up (LTFU; 14.7%). In Laos, four participants were excluded as they did not receive the intervention, and 34 participants (16.5%) were LTFU. In Thailand, 16 participants (7.3%) were LTFU. Final student sample sizes in Cambodia, Laos and Thailand were 202, 168 and 202, respectively. Sensitivity analyses compared baseline outcomes between participants LTFU and retained participants for age, sex and KAP responses in each study arm by country. No significant differences were found (*p* > 0.05). As such, only study participants who completed the KAP questionnaire at baseline and follow-up were included in this study. The demographic characteristics of the retained cohorts are presented in Table 4.

#### 3.1.2. Qualitative Studies

Parents and teachers at intervention schools were recruited at follow-up for qualitative data collection, yielding a total sample of 35 parents and seven teachers. This included five parents and one teacher in Cambodia, 15 parents and three teachers in Laos, and 15 parents and three teachers in Thailand. Student FGDs included a subset of student participants in each school (see Figure 1).

### 3.2. Quantitative Analyses

#### 3.2.1. Cambodia

There were no significant differences between intervention and control arms for sex (*p* = 0.487) or age (*p* = 0.449) (Table 4). There were no significant baseline differences between intervention and control mean scores for knowledge (8.7; 95% CI 4.5, 12.9 vs. 12.8; 95% CI 8.9, 16.8, *p* = 0.151) or practices (22.4; 95% CI 18.1, 26.7 vs. 26.4; 95% CI 22.1, 30.6, *p* = 0.196) (Table 5). Baseline attitude scores in the control arm were significantly higher by 7.3 percentage points (95% CI 1.4, 13.2, *p* = 0.033) compared to intervention (47.0; 95% CI 42.1, 52.0 vs. 39.7; 95% CI 36.9, 42.6, *p* = 0.016).

At follow-up, mean knowledge scores significantly increased in intervention (55.7; 95% CI 52.8, 58.6, *p* < 0.001) and control (40.6; 95% CI 35.0, 46.2, *p* < 0.001) arms. After adjusting for timepoint and study arm covariates, intervention mean knowledge and attitude scores were significantly higher than those of the control, increasing by 19.2 percentage points (95% CI 11.1, 27.4, *p* < 0.001) and 25.3 percentage points (95% CI 16.6, 34.0, *p* < 0.001), respectively (Table 6). Mean practice scores across study arms increased by 7.7 percentage points (95% CI 2.3, 13.1, *p* = 0.005) at follow-up. There was no significant intervention effect (*p* = 0.815).

#### 3.2.2. Laos

There were no significant differences between intervention and control arms for sex (*p* = 0.141) or age (*p* = 0.643) (Table 4).

There were no significant baseline differences between intervention and control arms in mean knowledge scores (24.3; 95% CI 20.5, 28.2 vs. 21.5; 95% CI 17.8, 25.2, *p* = 0.295) (Table 5), attitudes (42.3; 95% CI 37.2, 47.4 vs. 46.9; 95% CI 41.7, 52.0, *p* = 0.218), or practices (39.4; 95% CI 34.4, 44.3 vs. 34.9; 95% CI 30.4, 39.4, *p* = 0.195).

Mean knowledge scores significantly increased at follow-up in intervention (55.8; 95% CI 53.0, 58.5, *p* < 0.001) and control arms (33.9; 95% CI 28.1, 39.6, *p* = 0.002). After adjusting for timepoint and study arm covariates, intervention mean knowledge scores increased by 19.0 percentage points (95% CI 10.5, 27.6, *p* < 0.001) and attitudes by 14.2 percentage points (95% CI 5.5, 22.8, *p* = 0.001) (Table 6). There were no significant changes in practice scores (*p* > 0.05).

#### 3.2.3. Thailand

There was no significant difference in age between intervention and control arms (*p* = 0.414) (Table 4). The intervention arm had significantly more females than the control arm (*p* = 0.024). Bivariate and multivariate analyses were performed to assess the effect of sex on KAP scores. All results were non-significant (*p* > 0.05). As such, sex was not included in the main analyses.

At baseline, there were significant differences between intervention and control mean scores in knowledge (37.4; 95% CI 33.6, 41.2 vs. 11.7; 95% CI 8.0, 15.5, *p* < 0.001) and attitude (43.0; 95% CI 39.0, 47.0 vs. 29.1; 95% CI 25.3, 33.0, *p* < 0.001) (Table 5). There was no significant difference in mean practice scores (29.7; 95% 25.9, 33.5 vs. 34.3; 95% 30.2, 38.5, *p* = 0.106).

At follow-up, mean knowledge scores in intervention and control arms increased significantly by 14.8 percentage points (95% CI 10.7, 19.0, *p* < 0.001) and 38.1 percentage points (95% CI 32.5, 43.7, *p* < 0.001), respectively (Table 5). However, after accounting for timepoint and study arm covariates, improvements in intervention scores were lower than those in the control by 23.3 percentage points (95% CI −30.1, −16.5, *p* < 0.001) (Table 6). Intervention attitude scores significantly declined by 7.3 percentage points (95% CI −2.3, 12.3, *p* = 0.005). Conversely, control attitude scores significantly increased by 8.5 percentage points (95% CI 3.0, 14.0, *p* = 0.003). After adjusting for timepoint and intervention covariates, intervention attitude scores declined by 15.8 percentage points (95% CI −23.0, –8.5 *p* < 0.001) relative to the control. There were no significant effects on practices (*p* > 0.05).

### 3.3. Qualitative Analyses

#### 3.3.1. Cambodia

##### Liver Flukes and Fish Preparation

There was consensus among groups that liver flukes were an important issue. The teacher emphasised that messages should be shared with the broader community to improve awareness, noting that “[the video] reflects the reality of many communities, especially those in rural areas”. Parents also discussed the impact of the intervention on their own knowledge, with some stating that they only learned of liver fluke disease through their children. The importance of cooking fish thoroughly prior to consumption was emphasised in all groups, and the teacher and students also noted that as part of OV prevention, raw food scraps should not be fed to pets or other animals. Parents and children reported changing food preparation practices following the intervention.

##### General Hygiene

The importance of proper hygiene, including handwashing and safe defecation (i.e., using a latrine or toilet), for disease prevention was emphasised by the teacher, students and parents, although parents did note that open defecation sometimes occurred in the fields.

#### 3.3.2. Laos

##### OV Risk Factors

Unsafe fish consumption and open defecation as OV risk factors were common themes across discussions. Some parents learned of these risk factors through their children following the intervention, and others had been informed by previous village health campaigns or personal experience. There was also an understanding that other animals could also be infected by OV if they ate infected food, such as fish scraps. Several parents reported behavioural change following the intervention: “We don’t eat [raw fish]. There’s been a change. The kids stop us from eating raw food”.

##### Barriers

The main barriers to behavioural change were tradition and habits. Parents and teachers noted that while unsafe fish consumption in the village has declined, it did still occur, particularly at major events such as weddings. The impact of parents’ habits on children’s practices was also discussed as a barrier, with one teacher stating that “most students won’t eat raw food if their parents don’t give it to them. If their parents give them raw salads or raw meat, they will eat it. But if their parents cook it for them, they won’t eat raw food. This depends on the parents”.

#### 3.3.3. Thailand

##### Transmission and Symptoms

All discussions mentioned raw fish consumption as a transmission pathway for OV infection. Open defecation was also mentioned by one teacher, who noted that it may occur due to a lack of appropriate facilities. Students were aware of symptoms associated with OV infection, including nausea, fatigue, and risk of cancer in cases of long-term infection.

##### Knowledge Sharing

While parents were aware of this study, many did not know about its health messages in detail. Some children noted that they had not mentioned MGO to their parents. Nevertheless, parents supported the intervention as a tool for improving OV KAP. Teachers also emphasised the importance of sharing health messages to improve outcomes for families and the community.

## 4. Discussion

MGO achieved marked improvements in Cambodia and Laos for children’s self-reported knowledge and attitude scores in intervention arms, and this was further supported by the qualitative findings, indicating improved knowledge and behavioural change among students and parents. However, in Thailand, improvements in knowledge and attitude scores were far higher in the control arm, and attitude scores in the intervention group declined significantly (*p* < 0.001). The variation in scores between countries may be explained by socioeconomic differences. Cambodia and Laos are considered low–middle-income countries, whereas Thailand is classified as an upper-middle-income country [22]. As such, schoolchildren in Cambodia and Laos may have fewer available health education resources and therefore found the intervention more engaging and informative, leading to greater knowledge retention.

In Thailand, there was a 25-percentage-point difference in baseline knowledge scores between the intervention and control arms. All Thailand schools were selected from the same province with the intention of minimising sociodemographic and educational differences; however, it is possible that intervention schools had prior access to existing OV health education programmes [23], which could explain the difference in baseline knowledge scores between study arms. The monitoring of potential confounding suggested that control schools were aware of students’ low OV KAP scores; thus, additional school health education measures may have been implemented, which could explain marked improvements in knowledge and attitudes. Previous OV health education in the intervention schools could also account for the reduced intervention effect, as participants may have experienced learning fatigue and been less engaged by MGO. It is noteworthy that there was still a significant improvement in the knowledge score in the intervention arm.

While learning fatigue in intervention schools may be explained by OV education, the decline in attitude scores among participants in Thailand is cause for concern. Some studies have reported a positive association between learning fatigue and negative emotions, including apathy and melancholy [24,25]. Receiving high levels of OV education could therefore have a potential negative impact on OV-related attitudes and behaviours, as students may become less interested or inclined to practice safe OV practices. This could have severe consequences for long-term health outcomes; the monitoring of these impacts and further investigation is warranted.

There were no significant improvements in schoolchildren’s self-reported behaviour scores in any country. This may be because the study period was not long enough to capture changes in behaviour, which often occur gradually over time [26]. The lack of behavioural change may also be due to external factors. Open defecation typically occurred due to limited toilet facilities, rather than personal choice. Unsafe fish consumption persisted, often due to tradition or habit. Further, as children’s eating habits are largely governed by their families, it may be difficult for them to improve their eating practices independently. This underlies the importance of early health interventions to educate populations on infection risk and modify behaviour before habits are formed. However, in Cambodia and Laos, several parents reported they had stopped consuming raw or undercooked fish after learning about OV infection from their children. This suggests that the intervention did reduce OV risk-taking behaviour among some participants and their families.

An important finding of this study was the value of knowledge sharing. In Cambodia and Laos, children passed on key health messages to their parents, which in turn improved parents’ OV-associated knowledge and attitudes and, in some cases, behaviour. Across countries, participants supported MGO as an accessible and understandable tool for OV health education in schools and the broader community. In villages with existing health initiatives, MGO may be used to enhance existing health promotion strategies. Previous research conducted in Asia and sub-Saharan Africa [27,28,29] has demonstrated the effectiveness of school-based health education in improving students’ health KAP. The findings of this study support the role of school-based interventions in improving health outcomes in affected communities.

This study has several limitations. Due to the nature of the intervention, blinding was not possible. While standardised procedures were implemented both within and across countries, the results may have been impacted by observer bias. As suggested previously, knowledge of study arm allocation may have also contributed to contamination in control (and intervention) schools. Similarly, intervention interference may have affected the outcomes if participants were exposed to similar health education programmes. This could have reduced engagement in this study and the overall effectiveness of MGO. Baseline differences in KAP scores may have also confounded the study findings, although timepoint was included as a covariate in the regression models. The small number of clusters in some settings may have limited the generalisability of this study. Similarly, given the small qualitative sample size in some settings, it is possible that the findings are not representative of all participants. However, the similarities in qualitative responses between countries suggest that this was not the case. The follow-up period may have been insufficient to capture the long-term impact of MGO on OV KAP, although the extended follow-up period in Laos suggested that the intervention did have a sustained impact. We did not collect data on sociodemographic information or reasons for LTFU, but it is possible that residual confounding occurred, which could have affected KAP outcomes. Finally, reliance on self-reported practices may have resulted in measurement error. However, previous studies have supported the validity and reliability of self-reported data from children [30,31,32].

## 5. Conclusions

This study found that MGO significantly improved schoolchildren’s knowledge and attitudes surrounding OV and improved fish consumption practices among parents in Cambodia and Laos but not Thailand. These findings provide further support for the effectiveness of the MG health education package and its adaptability to different diseases and countries but with contextual differences impacting on intervention effectiveness across different country settings. Future research may include a scaling-up protocol of the MGO educational package for school delivery across the LMB, in coordination with other OV health initiatives, such as anthelmintic treatment and community engagement.

## Figures and Tables

**Table 1:** Delivery period by country.

Country	Baseline	Follow-Up
Cambodia	January 2024	November 2024
Laos	November 2023	December 2024
Thailand	September 2023	July 2024

**Table 2:** The details of the MGO health education package targeting OV in the intervention schools.

Timepoint	Educational Component	Aim
Initial delivery (following baseline KAP questionnaire)	MGO video shown once	Educate students on OV transmission, symptoms, treatment and prevention
	Classroom discussion guide distributed	
	MGO video shown a second time	Reinforce key messages
	Distribution of a comic pamphlet with key health messages	Provide a summary of key messages
	Classroom discussion	Confirm the understanding of health messages and answer students’ questions
	Drawing competition distributed (draw warning signs of OV infection) Best drawing receives an award	Reinforce key messages
Reinforcement session (6–8 weeks after intervention delivery)	MGO video shown once	Reinforce key messages
	Classroom discussion guide distributed	
	MGO video shown for a second time	Provide a summary of key messages
	Classroom discussion	Confirm the understanding of health messages and answer students’ questions
	Writing competition distributed (write actions that can be taken to prevent OV infection) Best essay receives an award	Reinforce key messages

KAP, knowledge, attitudes and practices; MGO, Magic Glasses Opisthorchiasis; OV, *Opisthorchis viverrini*.

**Table 3:** Question guides for the student and parent focus group discussions and teacher interviews.

Qualitative Data	Participants	Question Guide
Student FGD	4–6 students who received the intervention	Did you learn anything from the video? What did you learn? Do you think you/your family could carry out the actions described in the video? Why or why not? Do you think you/your family would want to carry out the actions described in the video? Why or why not?
Parent FGD	4–6 parents of children who received the intervention	Did you know about the research project at your children’s school? Did you learn about OV health education through your children? Do you think liver flukes are a major health problem in this region? If yes, did you learn this from your children? What’s the most important measure to fight liver flukes? Is this something you can do? What can you do to sustainably avoid liver flukes? Is this something you can do?
Teacher KII	One teacher from an intervention classroom	What do you think is the message of the video? How can you get liver fluke infections? Who do you think could benefit the most from watching this video? Do you think the messages in this cartoon are realistic (i.e., achievable)?

FGD, focus group discussion; KII, key informant interview; OV, *Opisthorchis viverrini*.

**Table 4:** Baseline characteristics of study participants, by country.

	Overall	Intervention	Control	*p*-Value
Cambodia	*n* = 202	*n* = 94	*n* = 108	
Male (%)	102 (50.5)	45 (47.9)	57 (52.8)	0.487
Mean age in years (SD)	10.0 (1.4)	10.1 (1.6)	9.9 (1.2)	0.449
Laos	*n* = 168	*n* = 91	*n* = 77	
Male (%)	62 (36.9)	29 (31.9)	33 (42.9)	0.141
Mean age in years (SD)	9.1 (1.0)	9.1 (1.0)	9.0 (1.1)	0.643
Thailand	*n* = 202	*n* = 107	*n* = 95	
Male (%)	102 (51.0)	46 (43.0)	56 (59.0)	0.024
Mean age in years (SD)	10.05 (1.0)	10.10 (1.0)	10.0 (1.0)	0.414

**Table 5:** Differences in scores of knowledge, attitude and practices between intervention and control schools, by country.

	Intervention Arm Mean Score (95% CI)	Control Arm Mean Score (95% CI)	Mean Difference (95% CI)	*p*-Value
Cambodia (*n* = 202)	*n* = 94	*n* = 108		
Knowledge				
Baseline	8.7 (4.5, 12.9)	12.8 (8.9, 16.8)	4.1 (−1.5, 9.9)	0.151
Follow-up	55.7 (52.8, 58.6)	40.6 (35.0, 46.2)	15.1 (8.5, 21.6)	<0.001
Mean difference (95% CI)	47.0 (41.9, 52.2)	27.8 (21.5, 34.1)		
*p*-value	<0.001	<0.001		
Attitudes				
Baseline	39.7 (36.9, 42.6)	47.0 (42.1, 52.0)	7.3 (1.4, 13.2)	0.016
Follow-up	66.5 (62.7, 70.3)	48.5 (42.2, 54.7)	18.0 (10.5, 25.6)	<0.001
Mean difference (95% CI)	26.8 (22.0, 31.5)	1.5 (−5.7, 8.6)		
*p*-value	<0.001	0.687		
Practices				
Baseline	22.4 (18.1, 26.7)	26.4 (22.1, 30.6)	4.0 (−2.1, 10.0)	0.196
Follow-up	31.1 (26.4, 35.7)	34.1 (29.2, 38.9)	3.0 (−3.7, 9.7)	0.373
Mean difference (95% CI)	8.7 (3.0, 14.3)	7.7 (2.0, 13.4)		
*p*-value	0.003	0.009		
Laos (*n* = 168)	*n* = 91	*n* = 77	Mean difference (95% CI)	
Knowledge				
Baseline	24.3 (20.5, 28.2)	21.5 (17.8, 25.2)	2.8 (−8.2, 2.5)	0.295
Follow-up	55.8 (53.0, 58.5)	33.9 (28.1, 39.6)	21.9 (15.8, 28.0)	<0.001
Mean difference (95% CI)	31.4 (26.6, 36.24)	12.4 (4.8, 20.0)		
*p*-value	<0.001	0.002		
Attitudes				
Baseline	42.3 (37.2, 47.4)	46.9 (41.7, 52.0)	4.5 (−2.7, 11.9)	0.218
Follow-up	53.8 (50.4, 57.2)	44.2 (40.1, 48.2)	9.6 (4.4, 14.8)	<0.001
Mean difference (95% CI)	11.5 (5.1, 17.8)	2.7 (−3.2, 8.6)		
*p*-value	<0.001	0.364		
Practices				
Baseline	39.4 (34.4, 44.3)	34.9 (30.4, 39.4)	4.5 (−2.3, 11.2)	0.195
Follow-up	44.1 (40.2, 48.0)	33.9 (28.5, 39.2)	10.2 (3.8, 16.7)	0.002
Mean difference (95% CI)	4.7 (−0.2, 9.7)	1.1 (−5.5, 7.6)		
*p*-value	0.059	0.749		
Thailand (*n* = 202)	*n* = 107	*n* = 95		
Knowledge				
Baseline	37.4 (33.6, 41.2)	11.7 (8.0, 15.5)	25.7 (20.3, 31.0)	<0.001
Follow-up	52.2 (48.7, 55.7)	49.8 (45.5, 54.2)	2.4 (−3.1, 7.9)	0.392
Mean difference (95% CI)	14.8 (10.7, 19.0)	38.1 (32.5, 43.7)		
*p*-value	<0.001	<0.001		
Attitudes				
Baseline	43.0 (39.0, 47.0)	29.1 (25.3, 33.0)	13.9 (8.3, 19.5)	<0.001
Follow-up	35.7 (31.7, 39.7)	37.6 (33.0, 42.2)	1.9 (−4.2, 7.9)	0.541
Mean difference (95% CI)	7.3 (−2.3, 12.3)	8.5 (3.0, 14.0)		
*p*-value	0.005	0.003		
Practices				
Baseline	29.7 (25.9, 33.5)	34.3 (30.2, 38.5)	4.6 (−1.0, 10.2)	0.106
Follow-up	31.5 (27.3, 35.7)	34.6 (29.7, 39.6)	3.1 (−3.3, 9.5)	0.337
Mean difference (95% CI)	1.8 (−3.3, 6.8)	0.3 (−4.6, 5.1)		
*p*-value	0.492	0.914		

CI, confidence interval.

**Table 6:** Generalised estimating equation model of study arm, time and intervention effect coefficients, by country.

	Cambodia			Laos			Thailand		
	Β (95% CI)	SE	*p*-Value	Β (95% CI)	SE	*p*-Value	Β (95% CI)	SE	*p*-Value
**Knowledge**									
Study arm ^1^ Intervention	−4.2 (−10.3, 1.9)	3.1	0.177	2.9 (−2.8, 8.5)	2.9	0.322	25.7 (20.3, 31.0)	2.7	<0.001
Timepoint ^2^ Follow-up	27.8 (22.2, 33.3)	2.8	<0.001	12.4 (6.1, 18.7)	3.2	<0.001	38.1 (33.2, 43.0)	2.5	<0.001
Study arm * timepoint ^3^ Intervention * follow-up	19.2 (11.1, 27.4)	4.2	<0.001	19.0 (10.5, 27.6)	4.4	<0.001	−23.3 (−30.1, −16.5)	3.5	<0.001
**Attitude**									
Study arm ^1^ Intervention	−7.3 (−14.0, −0.6)	3.4	0.033	−4.5 (−10.8, 1.7)	3.2	0.152	13.9 (8.1, 19.7)	2.9	<0.001
Timepoint ^2^ Follow-up	1.5 (−4.5, 7.4)	3.0	0.631	−2.7 (−9.0, 3.7)	3.2	0.406	8.5 (3.2, 13.8)	2.7	0.002
Study arm * timepoint ^3^ Intervention * follow-up	25.3 (16.6, 34.0)	4.4	<0.001	14.2 (5.5, 22.8)	4.4	0.001	−15.8 (−23.0, −8.5)	3.7	<0.001
**Practice**									
Study arm ^1^ Intervention	−4.0 (−10.3, 2.3)	3.2	0.216	4.5 (−2.1, 11.0)	3.3	0.180	−4.6 (−10.6, 1.3)	3.0	0.129
Timepoint ^2^ Follow-up	7.7 (2.3, 13.1)	2.8	0.005	−1.1 (−6.9, 4.7)	3.0	0.721	0.26 (−4.8, 5.3)	2.6	0.918
Study arm * timepoint ^3^ Intervention * follow-up	1.0 (−7.0, 8.9)	4.1	0.815	5.8 (−2.1, 13.7)	4.0	0.150	1.5 (−5.4, 8.4)	3.5	0.673

CI, confidence interval. ^1^ Reference category was control. ^2^ Reference category was baseline. ^3^ Reference category was control arm at follow-up. All regression models included timepoint and condition as covariates.

## Data Availability

The data that support the findings of this study will be made available upon reasonable request to the corresponding author.

## Supplementary Materials

The following supporting information can be downloaded at: https://www.mdpi.com/article/10.3390/tropicalmed11070174/s1. Supplementary Material S1. Published study protocol [14] (accessible at https://pmc.ncbi.nlm.nih.gov/articles/PMC11443236/). Supplementary Material S2. OV KAP questionnaire scoring “Magic Glasses Opisthorchiasis” cluster-randomised controlled trial [14]. Supplementary Table. Consort 2025 checklist of information [33].

## Abbreviations

FGD	Focus group discussion
KAP	Knowledge, attitudes and practices
KII	Key informant interview
LMB	Lower Mekong Basin
LTFU	Lost to follow-up
MG	Magic Glasses
MGO	Magic Glasses Opisthorchiasis
OV	*Opisthorchis viverrini*
