# Supplementary material for: The Impact of “The Magic Glasses Opisthorchiasis” on Schoolchildren’s Knowledge, Attitudes and Practices Surrounding Opisthorchis viverrini in the Lower Mekong Basin, a Cluster-Randomised Controlled Trial
Source: Trop Med Infect Dis. 2026 Jun 24;11(7):174. doi: 10.3390/tropicalmed11070174 (PMC13417138; doi:10.3390/tropicalmed11070174)
Supplement: Supplementary file 1 [file tropicalmed-11-00174-s001.zip › Supp 1. Published research protocol O'Connor et al..pdf]

Protocol

Examining the Acceptability of Helminth Education Packages "Magic Glasses Lower Mekong" and "Magic Glasses Opisthorchiasis" and Their Impact on Knowledge, Attitudes, and Practices Among Schoolchildren in the Lower Mekong Basin: Protocol for a Cluster Randomized Controlled Trial

Suji Y O'Connor[1], MPH; Mary Lorraine Mationg[1,2], PhD; Matthew J Kelly[1], PhD; Gail M Williams[3], PhD; Archie CA Clements[4], PhD; Banchob Sripa[5,6], PhD; Somphou Sayasone[7], PhD; Virak Khieu[8], PhD; Kinley Wangdi[9], PhD; Donald E Stewart[2,10], PhD; Sirikachorn Tangkawattana[6,11], PhD; Apiporn T Suwannatrai[6,12], PhD; Vanthanom Savathdy[7], MSc; Visal Khieu[8,13,14], MSc; Peter Odermatt[13,14], PhD; Catherine A Gordon[2], PhD; Sangduan Wannachart[6], MSc; Donald P McManus[2†], PhD, DSC; Darren J Gray[2], PhD

[1]National Centre for Epidemiology and Population Health, Australian National University, Acton, Australia
[2]Queensland Institute of Medical Research Berghofer Institute, Herston, Australia
[3]School of Public Health, University of Queensland, Herston, Australia
[4]Queen's University Belfast, Belfast, United Kingdom
[5]Department of Tropical Medicine, Faculty of Medicine, Khon Kaen University, Khon Kaen, Thailand
[6]Tropical Disease Research Center, Faculty of Medicine, Khon Kaen University, Khon Kaen, Thailand
[7]Lao Tropical and Public Health Institute, Ministry of Health, Vientiane, Lao People's Democratic Republic
[8]National Center for Parasitology, Entomology and Malaria Control, Ministry of Health, Phnom Penh, Cambodia
[9]HEAL Global Research Centre, Health Research Institute, Faculty of Health, University of Canberra, Bruce, Australia
[10]School of Medicine and Dentistry, Griffith University, Nathan, Australia
[11]Faculty of Veterinary Medicine, Khon Kaen University, Khon Kaen, Thailand
[12]Department of Parasitology, Faculty of Medicine, Khon Kaen University, Khon Kaen, Thailand
[13]Swiss Tropical and Public Health Institute, Allschwil, Switzerland
[14]University of Basel, Basel, Switzerland
[†]deceased

Corresponding Author:
Suji Y O'Connor, MPH
National Centre for Epidemiology and Population Health
Australian National University
62 Mills Road
Acton, 2601
Australia
Phone: 61 026125 2378
Email: suji.oconnor@anu.edu.au

Abstract

Background: Helminths are a major global health issue, impacting health, educational, and socioeconomic outcomes. Infections, often starting in childhood, are linked to anemia, malnutrition, cognitive deficit, and in chronic cases of *Opisthorchis viverrini* (OV), cholangiocarcinoma. The main control strategy for helminth infection is mass drug administration; however, this does not prevent reinfection. As such, prevention strategies are needed. The "Magic Glasses" is a school-based cartoon health education package that has demonstrated success in improving knowledge, attitudes, and practices (KAP) surrounding soil-transmitted helminths (STH) in China and the Philippines. This study is designed to assess the acceptability and impact of the 2 new versions of the Magic Glasses targeting STH and OV designed for the Lower Mekong audience in Cambodia, Lao People's Democratic Republic (PDR), and Thailand.

XSL•FO
RenderX

Objective: The objective of this study is to evaluate the acceptability of the “Magic Glasses Lower Mekong” and “Magic Glasses Opisthorchiasis” education packages among schoolchildren in the Lower Mekong Basin, and the impact of these education packages on students’ KAP surrounding STH and OV, respectively.

Methods: Schoolchildren will be recruited into a cluster randomized controlled trial with intervention and control arms in rural schools in Cambodia, Lao PDR, and Thailand. Schoolchildren’s initial acceptability of the intervention will be evaluated using an adapted questionnaire. Sustained acceptability will be assessed at 9-month follow-up through focus group discussions with students and interviews with teachers. Impact will be evaluated by KAP questionnaires on STH and OV. KAP questionnaires will be administered to children at baseline and at follow-up. Indirect impact on parents' KAP of OV and STH will be assessed through focus group discussions at follow-up.

Results: The trial is in progress in Lao PDR and Thailand and is expected to commence in Cambodia in January 2024. The results of the study are expected to be available 18 months from the start of recruitment. We hypothesize that participants enrolled in the intervention arm of the study will have higher KAP scores for STH and OV, compared with the participants in the control arm at follow-up. We expect that students will have initial and sustained acceptability of these intervention packages.

Conclusions: This trial will examine the acceptability of the “Magic Glasses Opisthorchiasis” and “Magic Glasses Lower Mekong” interventions and provide evidence on the effectiveness of the “Magic Glasses” on KAP related to OV and STH among schoolchildren in the Lower Mekong Basin. Study results will provide insight on acceptability and impact indicators and inform a scaling up protocol for the “Magic Glasses” education packages in Cambodia, Lao PDR, and Thailand.

Trial Registration: Australian New Zealand Clinical Trials Registry ACTRN12623000271606; https://www.anzctr.org.au/Trial/Registration/TrialReview.aspx?id=385315&isReview=true

International Registered Report Identifier (IRRID): DERR1-10.2196/55290

(JMIR Res Protoc 2024;13:e55290) doi: 10.2196/55290

KEYWORDS

attitude; child; health education; helminths; knowledge; practices; Opisthorchis viverrini; randomized controlled trial; schools; Ascaris lumbricoides; Trichuris trichiura; hookworm; Magic Glasses

Introduction

Background

Helminthiases, or infections caused by parasitic worms, are a major global health concern [1]. Communities impacted by poverty and located in tropical regions, such as the Lower Mekong Basin (LMB; a region including Cambodia, Lao People’s Democratic Republic [Lao PDR], and northeast Thailand) are particularly vulnerable to such infections. The most common helminthiases in the LMB are soil-transmitted helminths (STH)—including *Ascaris lumbricoides*, *Trichuris trichiura*, and hookworm (*Ancylostoma* spp. and *Necator americanus*)—and *Opisthorchis viverrini* (OV), commonly known as the Southeast Asian liver fluke [2]. Reported prevalence of STH infections have reached 74% (227/308) [3], 83% (134/162) [4], and 28% (24/85) [5], in Cambodia, Lao PDR, and Thailand, respectively. OV is estimated to affect 10 million people in the LMB, the highest reported burden globally [6,7]. Several studies have reported high incidence of helminthiases among preschool- and school-aged children (SAC), often with high infection intensity [2,8-11]. Kaewpitoon et al [2] reported overall STH prevalence of 11.88% (233/1957) among SAC in the LMB in 2015; however, in some areas, prevalence reached 16.08% (152/945). Given the low sensitivity of the Kato-Katz thick smear technique used (particularly for low intensity infections), it is also possible that infection prevalence in the region was underestimated [12]. While there is limited research on OV burden in children, Yoshida et al [13] reported an OV infection rate of 39% (64/164) among children in secondary school in Vientiane, Lao PDR. Helminthiases are associated with stunting, malnutrition, anemia, chronic pain, fatigue, and cognitive deficit [14-17]. In addition, chronic OV infections are causally linked to cholangiocarcinoma, a cancer of the bile duct [18]. Transmission route varies by helminth type: STH is typically transmitted via contact with egg-infected soil, such as consumption of contaminated fruit or vegetables, or playing in contaminated outdoor spaces [19]. OV is transmitted via ingestion of raw or undercooked infected freshwater cyprinid fish [20]. The primary control strategy for STH and OV infection is mass drug administration (MDA); STH infection is typically treated with benzimidazole anthelmintics [19]. Treatment efficacy varies against species: cure rates exceed 90% for *Ascaris*, 50% for hookworm species [21], and 25% for *Trichuris* [22]. The recommended treatment for OV is praziquantel [23]. Several studies have found praziquantel to be highly efficacious against OV infection, with cure rates exceeding 90% [24,25]. However, while MDA is effective in treating helminthiases, it does not prevent reinfection. As such, further measures such as education on helminth control and prevention are needed.

Children are an important group for targeted behavioral and educational interventions. Between the ages of 5 and 12 years, children undergo major cognitive growth, including the formation of beliefs and attitudes [26,27]. Health education during this period can inform long-term habits and behaviors [26-28]. SAC are also a well-placed group for intervention delivery, as schools are institutions trusted by children and their families, and can facilitate delivery to a large population, as recognized in World Health Organization’s (WHO) Global School Health Initiative [29].

XSL•FO
RenderX

The "Magic Glasses" is a school-based cartoon health education package that has demonstrated success in improving STH knowledge and attitudes, improving water, sanitation, and hygiene (WASH) practices and reducing disease burden in affected communities [30,31]. The intervention is designed to be relatable and engaging for children, with the cartoon set in the local context, and the protagonists being schoolchildren who learn about STH transmission, symptoms, treatment, and prevention as they explore their community [30,31]. The original "Magic Glasses" in China achieved major improvements in self-reported STH knowledge, attitudes and behaviors, as measured by a knowledge, attitudes, and practices (KAP) questionnaire: the intervention group scored 24.9 percentage points higher than the control after adjusting for baseline scoring (95% CI 23.4-26.4; $P<.001$) [31]. Overall KAP scores were inversely correlated with STH infection, with uninfected students scoring 9.9 percentage points higher than infected students (95% CI 5.8-14.0; $P<.001$) [31]. With respect to infection burden, the intervention led to a 50% decrease in STH incidence among intervention schools (odds ratio 0.5, 95% CI 0.35-0.70; $P<.001$) [31]. From 2016 to 2017, the "Magic Glasses" educational package was adapted for the Philippines, a country with estimated 33% STH prevalence [30]. "Magic Glasses Philippines" led to significant improvements in knowledge and behavior among intervention schools scoring 5.5 percentage points higher than control schools in the knowledge component of the KAP questionnaire (56.8, 95% CI 55.7-57.9 vs 51.5, 95% CI 50.3-53.7; $P<.001$) and 1.1 percentage points higher in the behavioral component (87.2, 95% CI 86.6-87.9 vs 86.1, 95% CI 85.4-86.8; $P=.002$) [30]. With respect to disease burden, the intervention achieved a 60% reduction in the odds of any STH infection in intervention schools with a baseline prevalence <15% (adjusted odds ratio 0.4, 95% CI 0.20-0.70; $P<.001$), although it is important to note that the intervention did not significantly reduce odds of infection when higher prevalence schools were included [30]. Following the success of the "Magic Glasses Philippines" trial, the intervention is now being recommended for roll-out across the Philippines as part of the school curriculum.

The present study is designed to assess the acceptability of 2 new "Magic Glasses" cartoons, developed for the LMB, and their impact on schoolchildren's self-reported KAP of STH and OV. Several studies have identified acceptability as a key indicator for initial and sustained intervention success [32-36]. However, there remains a lack of consistency among acceptability measures and definitions in public health research. Sekhon et al [37] developed the Theoretical Framework of Acceptability (TFA), and defined acceptability as "a multi-faceted construct that reflects the extent to which people delivering or receiving a healthcare intervention consider it to be appropriate, based on anticipated or experienced cognitive and emotional responses to the intervention" [37]. The TFA outlined 7 domains of acceptability, "affective attitude, burden, perceived effectiveness, ethicality, intervention coherence, opportunity costs, and self-efficacy" [37]. In order to conduct a robust assessment of all the domains of acceptability, the acceptability of the intervention will be assessed using mixed methods. Quantitatively using an adapted version of the TFA questionnaire [38] and qualitatively using focus group discussions (FGDs) with schoolchildren and key informant interviews (KIIs) with teachers. The KAP questionnaire will be administered at baseline and follow-up to assess the impact of the intervention, and the intervention coherence domain of acceptability.

Aims

While previous Magic Glasses studies have demonstrated the impact of the cartoon in improving KAP surrounding helminths, a more detailed assessment of acceptability is required. Firstly, this study aims to determine whether the "Magic Glasses" OV and STH video education packages will be acceptable to schoolchildren and their teachers in LMB. Secondly, this study will be the first "Magic Glasses" to target OV, in addition to STH. Finally, this study aims to determine the impact of the "Magic Glasses" cartoons on students' KAP on OV and STH infection.

Methods

Trial Design

The trial is a cluster randomized controlled trial (cRCT) with 2 arms, intervention and control. As the intervention will be delivered through schools, a cRCT design was chosen for practical reasons and to minimize contamination. Each school participating in the trial is a "cluster." Schools will be randomly allocated to intervention and control. Schools in the intervention arm will receive the "Magic Glasses" education package developed for LMB, consisting of "Magic Glasses Opisthorchiasis" (MGO) and "Magic Glasses Lower Mekong" (MGLM), in addition to the standard health education activities in accordance with local government guidelines. Schools in the control arm of the study will receive the standard health education activities only in accordance with local government guidelines.

Ethical Considerations

The study will be conducted in accordance with the ethical principles of the Australian National Statement on Ethical Conduct in Human Research [39]. Prior to data collection, an information sheet detailing the purpose of the study, potential risks, research contact information, and informed consent form in the local language will be distributed to parents and caregivers. Only schoolchildren who have provided informed assent and parental consent will be included in the study. Participants will not receive compensation for their involvement in the study but will be able to withdraw at any time. In the case that a participant does withdraw from the study, their responses will not be used in analyses. All quantitative data will be collected on paper questionnaires. Qualitative data (KIIs, FGDs) will be recorded using audiotapes and transcribed. To protect participant privacy, all responses will be deidentified prior to data analyses.

The study protocol was submitted to and received ethical approval from the Australian National University Human Ethics Committee (approval number 2022/507), the Cambodia Ministry of Health National Ethics Committee for Health Research (N197NECHR dated June 27, 2023), the Lao PDR Ministry of Health National Ethics Committee for Health Research, and

XSL•FO
RenderX

Khon Kaen University Ethics Committee for Human Research (HE651147, record number 4.2.03:16/2565). The study is registered at Australian New Zealand Clinical Trials Registry (ANZCTR): ACTRN12623000271606.

Setting

The trial will be conducted in rural primary schools in Preah Vihear Province, Cambodia; Savannakhet Province, Lao PDR, and Khon Kaen Province, Thailand. To maximize homogeneity within each country, schools will be recruited from the same district where possible. To minimize the risk of contamination, all control and intervention schools will be at least 3 km apart.

Participants

A total of 684 schoolchildren in Grades 3-6 from randomly selected schools in Cambodia, Lao PDR, and Thailand will be recruited into the study cohort and followed up approximately 9 months after baseline. During the follow-up period, 1 teacher and 3-5 parents will be recruited from each intervention school.

Eligibility Criteria

Among the recruited schools, children across Grades 3-6 who provided consent and assent will be eligible for this study.

Consent and Recruitment

Based on local expertise on school student numbers in each country, approximately 6 schools will be selected in each country to achieve a total sample size of 228 per country. Prospective schools will be selected in consultation with the local departments of education. Once schools are identified, permission to conduct the study will be sought from the principal of each selected school. Teachers and parents or legal guardians of prospective participants will then receive an information sheet outlining the procedure and purpose of the study and contact information, and a consent form. On receiving the written parental or guardian consent, the aims, process, and outcome of study will be explained to prospective student participants and their written assent will be sought. Only schoolchildren who have provided informed assent and parental consent will be included in the study. Teachers and parents will be recruited a month before follow-up. As part of the recruitment process, an information sheet will be provided and informed consent will be sought. Once written consent is obtained, teachers and parents will be recruited into the study.

Randomization

Schools within each country will be randomly allocated to control or intervention arm using Microsoft excel (Microsoft 365) RAND function. Allocation will remain for the duration of the project.

Blinding

Blinding will not be possible in this cRCT. Due to the nature of the intervention, participants will be aware of whether they are in the intervention or control arm.

Intervention

The “Magic Glasses” education package will be comprised of two animated cartoon packages developed specifically for the Lower Mekong setting: (1) MGO, an educational cartoon addressing OV infection, including transmission, symptoms, treatment, and prevention, and (2) MGLM, an educational cartoon addressing STH infection, including transmission, symptoms, treatment, and prevention. Each package includes an educational cartoon, an accompanying comic pamphlet with key messages and images from the cartoon, and engaging reinforcement activities for students (drawing competition and essay competition).

Cartoon Production

The MGO and MGLM interventions were adapted from previous Magic Glasses interventions [30,31], informed by literature reviews on risk factors and KAP regarding STH and OV in LMB. In line with previous “Magic Glasses” cartoons, the health messages of the cartoon were informed by developmental psychology theories including the Theory of Planned Behavior [40] and Social Cognitive Theory [41] to promote engagement and effectiveness with schoolchildren. In order to ensure that the cartoon style and backgrounds are relevant to SEA audiences, the cartoons were produced by 2D animation company, a Thai animation company based in Khon Kaen. Once the Thai versions of MGO and MGLM were finalized, Cambodian and Lao versions of each cartoon were adapted to include culturally appropriate attire and local languages (see Figure 1). The dialogues for each country’s cartoons were then recorded by voice actors in Thai, Khmer, and Lao. In total, 6 cartoons were developed.

Figure 1. Cambodia, Lao, and Thai versions (left to right) of the Doctor character in the “Magic Glasses Opisthorchiasis” and “Magic Glasses Lower Mekong” educational cartoons.

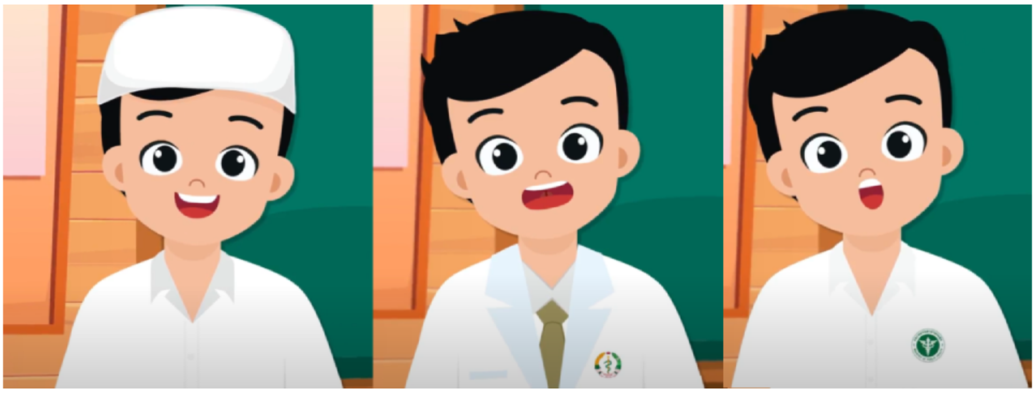

XSL•FO
RenderX

Comic Pamphlet

A comic pamphlet was developed for OV and STH summarizing the key health messages for each cartoon. The images included in the OV and STH comic pamphlet were taken from the health message summary at the end of the MGO and MGLM cartoon, respectively.

Study Procedures

Study commencement will be staggered across Thailand, Cambodia, and Lao PDR, based on the school periods in each country. Prior to study commencement and intervention delivery, all study materials will be translated into the local language by the local research team in each country. For anticipated study dates in each country, see Multimedia Appendix 1.

Baseline KAP Assessment

Prior to intervention delivery, all consenting participants in Grades 3-6 in control and intervention schools will complete 2 questionnaires on KAP surrounding OV and STH. The first questionnaire will include questions on demographic information and students' knowledge of OV, including general knowledge, transmission, symptoms, and treatment; attitudes and health education, and behavior. The second questionnaire will follow the same format to assess students' knowledge of STH. The STH KAP questionnaire is a validated questionnaire used in previous "Magic Glasses" projects [30,31]. The OV KAP questionnaire was adapted from the STH KAP and reviewed by disease experts on the project prior to finalization (see Multimedia Appendices 2 and 3 for questionnaires). Paper questionnaires will be administered in classrooms by research staff and completed by students individually. To minimize confusion between OV and STH, baseline questionnaires for the respective infections will be administered on separate days.

"Magic Glasses Opisthorchiasis" Delivery and Acceptability Assessment

In intervention schools, the OV-baseline KAP assessment and OV intervention will be administered on the same day. Once OV-baseline KAP assessment has been completed, the MGO intervention package will be administered. MGO will be presented to schoolchildren and teachers in their classroom twice. Once the cartoon has been shown, schoolchildren will receive a comic pamphlet with the key messages of the cartoon. A classroom discussion will then be held to go over the messages and clarify any confusion among the students. Following the classroom discussion, an acceptability questionnaire will be administered to assess students' initial acceptability of the cartoon. As with the KAP assessment, paper questionnaires will be administered in classrooms by research staff and completed by students individually. The acceptability questionnaire will contain multiple-choice and open-ended questions (see Multimedia Appendices 4 and 5). The questionnaire was adapted from the TFA [38] to include age-appropriate language for the schoolchildren, and specific questions on OV. Once the acceptability assessment is completed, the homework drawing competition will be explained: students will be asked to draw warning signs of OV infection, using the messages from the cartoon and comic pamphlet as a guide.

A reinforcement session will be held 6-8 weeks after the first presentation of MGO. The cartoon will be replayed twice to the student cohort, followed by a classroom discussion and explanation of the homework essay competition. Students will be asked to identify people who are at risk of liver fluke infection and write down why they are at risk, and how they can avoid liver flukes.

"Magic Glasses Lower Mekong" and Acceptability Assessment

In intervention schools, delivery of the MGLM intervention package will take place on the same day as the STH KAP questionnaire, once baseline data collection is complete. The MGLM package will follow the same delivery procedures used for the MGO intervention package.

Follow-Up

All participants in control and intervention schools will be recruited for follow-up approximately 9 months after baseline. Participants will complete the same questionnaires for OV and STH to assess the impact of the interventions on KAP for the respective diseases. KAP scores will also provide insight on acceptability domains of intervention coherence and perceived effectiveness. In intervention schools, impact and acceptability will also be assessed using qualitative measures. FGDs will be conducted with a subset of schoolchildren in the original cohort to discuss the sustained acceptability and effectiveness of each cartoon. FGDs will also be conducted with parents of participants to investigate the indirect impact of the MGO and MGLM intervention packages on parents' knowledge of OV and STH, respectively. KIIs will be held with teachers to discuss the acceptability and impact of the cartoons on health-related attitudes and behavior.

Follow-up assessments will employ the same delivery timing as used at baseline and intervention delivery to ensure consistency and minimize confusion between OV and STH.

The trial study design is shown in Figure 2. See Multimedia Appendices for anticipated delivery times for each country.

XSL•FO
RenderX

Figure 2. Trial Profile: Expected study enrollment and timeline diagram for the “Magic Glasses Opisthorchiasis” and “Magic Glasses Lower Mekong” acceptability and impact cluster randomized controlled trial. FGD: focus group discussion; KAP: knowledge, attitudes, and practices; KII: key informant interviews; MGLM: Magic Glasses Lower Mekong; MGO: Magic Glasses Opisthorchiasis.

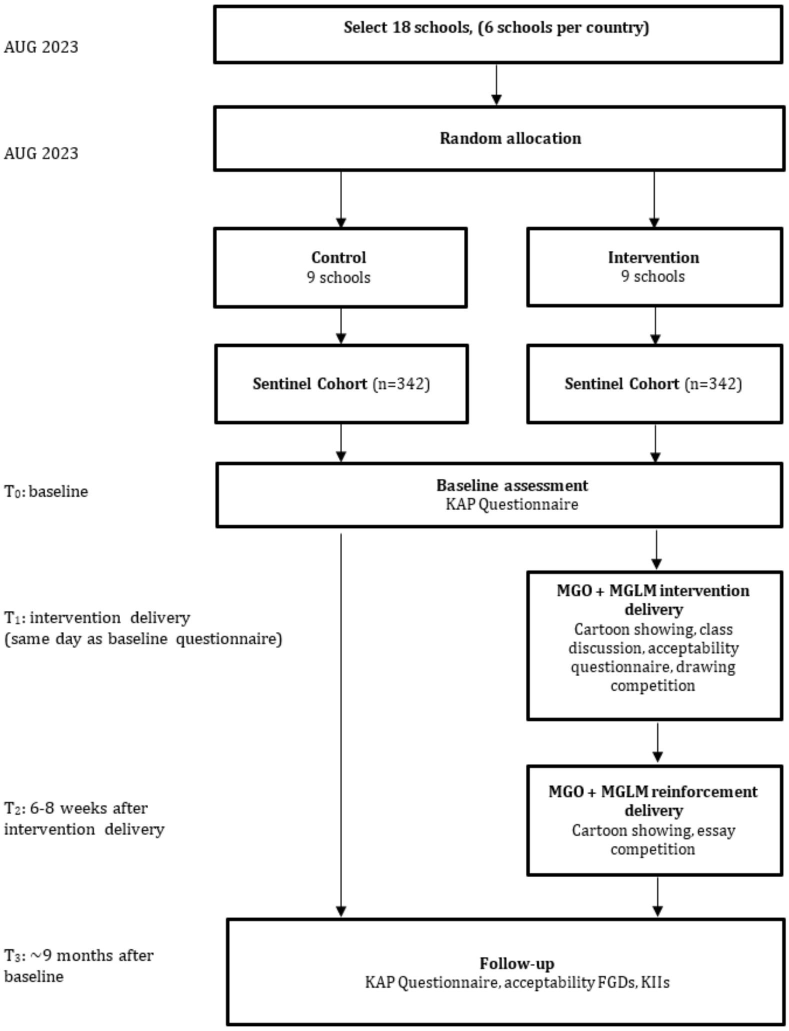

Data Analysis

Quantitative

Descriptive statistics will be computed to ascertain variable distributions.

Normality of distribution will be checked using the Shapiro-Wilk test, with significance set to $P<.05$ (2-sided). In acceptability analyses, negatively worded statements will be reverse scored to align the direction of the scale. Spearman correlation coefficient will be used to assess criterion validity among different questionnaire items. Exploratory factor analysis with oblique rotation will then be performed to assess construct validity. The primary outcome of the study is KAP scores for OV and STH.

OV and STH KAP scores will be analyzed separately. KAP scores for each questionnaire will be developed by calculating the total points for knowledge, attitude, and behavior for each student and expressed as a percentage score. Changes in self-reported KAP scores will be analyzed using linear regression. To assess the differences in KAP between control and intervention schools at baseline and follow-up, generalized-estimating equation models will be used to account for clustering within schools and repeated KAP assessment for participants. Potential confounders including age and sex will be incorporated in the model. Spearman correlation coefficient will be used to estimate correlations among acceptability, knowledge, attitudes, and self-reported behavior. Chi-square tests will also be used to measure associations between KAP and acceptability.

Qualitative

Content analysis [42] and thematic analysis [43] will be undertaken to determine the major categories in the qualitative data (acceptability FGDs and KIIs). Recordings will be translated verbatim to English and transcribed. An inductive approach will then be employed to analyze the recordings of FGDs and KIIs. Codes will be generated as part of the initial review process and entered into a standardized extraction table. Coded data will then be examined for key themes. Themes will be categorized using thematic maps to identify key and shared concepts across themes. Once organized, themes will be reviewed and defined, and key extracts identified. Acceptability and KAP qualitative data will first be examined separately, and then reviewed together to identify shared themes.

XSL•FO
RenderX

All statistical analyses will be performed using IBM SPSS (IBM SPSS Inc), Stata (StataCorp) or SAS software (SAS Institute Inc). Microsoft Excel, and NVivo (QSR International) will be used for qualitative analyses.

Power

The sample size was calculated to achieve 80% power, assuming baseline knowledge of 30 percentage points (as reported in the original "Magic Glasses" intervention by Bieri et al [31]) and a design effect of 2 to account for cluster sampling. Accounting for 10% attrition, it was estimated that a total of 227 participants would be needed to detect a difference of 5 percentage points in knowledge between intervention and control groups. As such, we will aim to enroll a total of 228 schoolchildren in years 3-5 (114 in intervention arm, 114 in control arm) in each country.

Results

The trial is in progress in Lao PDR and Thailand and is expected to commence in Cambodia in January 2024. Study results are expected to be available 18 months from the start of recruitment and will be submitted for publication thereafter.

Discussion

This study was designed to assess the acceptability of the MGO and MGLM cartoon packages, and to evaluate the impact of these packages on schoolchildren's KAP on OV and STH infection, respectively, in Cambodia, Thailand and Lao PDR. The expected outcomes are initial and sustained acceptability of the MGO and MGLM cartoon packages and improved KAP on OV and STH infection among intervention students. Several studies have outlined the importance of child-targeted health education interventions to improve health knowledge and behavior and prevent disease [26-28]. This is particularly important for populations at-risk of diseases such as OV and STH, which can have long-term health and developmental impacts [14-18]. Previous "Magic Glasses" studies have demonstrated the effectiveness of a school-based educational cartoon in improving KAP among schoolchildren in China and Philippines, and reducing the STH burden [30,31]. The "Magic Glasses Philippines" demonstrated the generalizability of "Magic Glasses" to other regions with endemic infectious diseases [30]. This trial will provide insight on the translatability of "Magic Glasses" to other infectious diseases with different methods of transmission and prevention. In addition, this will be the first study to formally assess the acceptability of "Magic Glasses" intervention.

This study does have some limitations. First, this study relies on self-reported data from children, which could lead to measurement error. Schoolchildren's self-reported OV and STH behavior in particular, may be misrepresented due to social desirability bias. However, several studies have supported child self-reporting as a valid and reliable measure in health research [44-46]. Second, while children receiving the intervention will learn how to prevent OV transmission by adequately cooking freshwater cyprinid fish, it is possible that they will not be able to do this, due to parental control over household food preparation. For this reason, we will also investigate the indirect impact of the intervention on parents' KAP relating to OV and STH infection. Second, due to the nature of the study, it was not possible to blind participants or research staff. Therefore, it is possible that nonblinding bias could occur.

It is expected that the acceptability and KAP findings will provide evidence for the scalability of the "Magic Glasses: Lower Mekong" and "Magic Glasses: Opisthorchiasis" interventions for Cambodia, Lao PDR, and Thailand and more broadly, provide further support for the "Magic Glasses" intervention. These findings have implications for the expansion of the "Magic Glasses" to other infectious diseases and countries, as a novel, engaging and cost-effective tool in school-based health education.

Acknowledgments

This research is supported by an e-Asia grant from the Australian National Health and Medical Research Council (GNT1184406). KW is funded by Australian National Health and Medical Research Council 2021 Investigator Grant (2008697). SYO is supported by Australian Government Research Training Program Domestic Scholarship.

Data Availability

Data will be archived in an encrypted server at the QIMR Berghofer Medical Research Institute. The deidentified data sets generated over the course of this study will be made available from the corresponding author on reasonable request.

Authors' Contributions

DJG, DPM, and GMW conceived and designed the "Magic Glasses" intervention. SYO, MLM, MJK, KW, and DJ designed the research. SYO drafted the first version of the manuscript with input from all authors. DJG, MLM, MJK, and GMW provided guidance on the design as well as data analysis planning. SYO, MLM, MJK, GMW, ACAC, BS, SS, BK, KW, DES, ST, ATS, VS, Virak K, PO, CAG, SW, and DJG reviewed the manuscript. All authors approved the manuscript.

Conflicts of Interest

None declared.

XSL•FO
RenderX

Multimedia Appendix 1

Anticipated delivery dates for baseline, intervention delivery, reinforcement and follow-up for the "Magic Glasses Lower Mekong" and "Magic Glasses Opisthorchiasis" cluster randomized controlled trial in Cambodia, Lao PDR, and Thailand.
[DOCX File , 23 KB-Multimedia Appendix 1]

Multimedia Appendix 2

Opisthorchis viverrini knowledge, attitudes, and practices questionnaire for the "Magic Glasses Lower Mekong" and "Magic Glasses Opisthorchiasis" cluster randomized controlled trial.
[DOCX File , 29 KB-Multimedia Appendix 2]

Multimedia Appendix 3

Soil-transmitted helminths knowledge, attitudes, and practices questionnaire for the "Magic Glasses Lower Mekong" and "Magic Glasses Opisthorchiasis" cluster randomized controlled trial.
[DOCX File , 35 KB-Multimedia Appendix 3]

Multimedia Appendix 4

Magic Glasses Opisthorchiasis acceptability questionnaire for the "Magic Glasses Lower Mekong" and "Magic Glasses Opisthorchiasis" cluster randomized controlled trial.
[DOCX File , 27 KB-Multimedia Appendix 4]

Multimedia Appendix 5

Magic Glasses Lower Mekong acceptability questionnaire for the "Magic Glasses Lower Mekong" and "Magic Glasses Opisthorchiasis" cluster randomized controlled trial.
[DOCX File , 28 KB-Multimedia Appendix 5]

References

1. Wright JE, Werkman M, Dunn JC, Anderson RM. Current epidemiological evidence for predisposition to high or low intensity human helminth infection: a systematic review. Parasit Vectors. 2018;11(1):65. [FREE Full text] [doi: 10.1186/s13071-018-2656-4] [Medline: 29382360]
2. Kaewpitoon SJ, Loyd RA, Kaewpitoon N. Home healthcare program for soil-transmitted helminthiasis in schoolchildren along the mekong river Basin. J Med Assoc Thai. 2015;98 Suppl 4:S1-S8. [Medline: 26201127]
3. Sinuon M, Anantaphruti MT, Socheat D. Intestinal helminthic infections in schoolchildren in Cambodia. Southeast Asian J Trop Med Public Health. Jun 2003;34(2):254-258. [Medline: 12971545]
4. Conlan JV, Khamlome B, Vongxay K, Elliot A, Pallant L, Sripa B, et al. Soil-transmitted helminthiasis in Laos: a community-wide cross-sectional study of humans and dogs in a mass drug administration environment. Am J Trop Med Hyg. 2012;86(4):624-634. [FREE Full text] [doi: 10.4269/ajtmh.2012.11-0413] [Medline: 22492147]
5. Kache R, Phasuk N, Viriyavejakul P, Punsawad C. Prevalence of soil-transmitted helminth infections and associated risk factors among elderly individuals living in rural areas of southern Thailand. BMC Public Health. Dec 07, 2020;20(1):1882. [FREE Full text] [doi: 10.1186/s12889-020-09986-7] [Medline: 33287781]
6. Ziegler AD, Petney TN, Grundy-Warr C, Andrews RH, Baird IG, Wasson RJ, et al. Dams and disease triggers on the lower Mekong river. PLoS Negl Trop Dis. 2013;7(6):e2166. [FREE Full text] [doi: 10.1371/journal.pntd.0002166] [Medline: 23853695]
7. Sripa B, Bethony JM, Sithithaworn P, Kaewkes S, Mairiang E, Loukas A, et al. Opisthorchiasis and opisthorchis-associated cholangiocarcinoma in Thailand and Laos. Acta Trop. 2011;120 Suppl 1(Suppl 1):S158-S168. [FREE Full text] [doi: 10.1016/j.actatropica.2010.07.006] [Medline: 20655862]
8. Weatherhead JE, Hotez PJ, Mejia R. The global state of helminth control and elimination in children. Pediatr Clin North Am. 2017;64(4):867-877. [FREE Full text] [doi: 10.1016/j.pcl.2017.03.005] [Medline: 28734515]
9. Ojja S, Kisaka S, Ediau M, Tuhebwe D, Kisakye AN, Halage AA, et al. Prevalence, intensity and factors associated with soil-transmitted helminths infections among preschool-age children in Hoima district, rural western Uganda. BMC Infect Dis. 2018;18(1):408. [FREE Full text] [doi: 10.1186/s12879-018-3289-0] [Medline: 30119650]
10. González Quiroz DJ, Agudelo Lopez SDP, Arango CM, Acosta JEO, Bello Parias LD, Alzate LU, et al. Prevalence of soil transmitted helminths in school-aged children, Colombia, 2012-2013. PLoS Negl Trop Dis. 2020;14(7):e0007613. [FREE Full text] [doi: 10.1371/journal.pntd.0007613] [Medline: 32678821]
11. Mationg M, Tallo V, Williams G, Gordon C, Clements A, McManus D, et al. The control of soil-transmitted helminthiases in the Philippines: the story continues. Infect Dis Poverty. 2021;10(1):85. [FREE Full text] [doi: 10.1186/s40249-021-00870-z] [Medline: 34118990]

XSL•FO
RenderX

12. Bärenbold O, Raso G, Coulibaly JT, N'Goran EK, Utzinger J, Vounatsou P. Estimating sensitivity of the kato-katz technique for the diagnosis of schistosoma mansoni and hookworm in relation to infection intensity. PLoS Negl Trop Dis. 2017;11(10):e0005953. [FREE Full text] [doi: 10.1371/journal.pntd.0005953] [Medline: 28976979]
13. Yoshida I, Horie O, Akkhavong K. Predictors of hookworm and infection among adolescents in urban Laos: a cross-sectional study. Res Rep Trop Med. 2019;10:31-41. [FREE Full text] [doi: 10.2147/RRTM.S199577] [Medline: 31191083]
14. King CH. Chapter two - helminthiasis epidemiology and control: scoring successes and meeting the remaining challenge. In: Keiser J, editor. Advances in Parasitology. Cambridge, MA. Academic Press; 2019.
15. Ezeamama AE, Friedman JF, Olveda RM, Acosta LP, Kurtis JD, Mor V, et al. Functional significance of low-intensity polyparasite helminth infections in anemia. J Infect Dis. 2005;192(12):2160-2170. [doi: 10.1086/498219] [Medline: 16288383]
16. Gyorkos TW, Gilbert NL. Blood drain: soil-transmitted helminths and anemia in pregnant women. PLoS Negl Trop Dis. 2014;8(7):e2912. [FREE Full text] [doi: 10.1371/journal.pntd.0002912] [Medline: 25010736]
17. Brooker S, Hotez PJ, Bundy DA. Hookworm-related anaemia among pregnant women: a systematic review. PLoS Negl Trop Dis. 2008;2(9):e291. [FREE Full text] [doi: 10.1371/journal.pntd.0000291] [Medline: 18820740]
18. Brindley PJ, da Costa JMC, Sripa B. Why does infection with some helminths cause cancer? Trends Cancer. 2015;1(3):174-182. [FREE Full text] [doi: 10.1016/j.trecan.2015.08.011] [Medline: 26618199]
19. World Health Organization. Soil-transmitted helminthiases. 2022. URL: https://www.who.int/health-topics/soil-transmitted-helminthiases [accessed 2023-10-11]
20. Vonghachack Y, Odermatt P, Taisayyavong K, Phounsavath S, Akkhavong K, Sayasone S. Transmission of opisthorchis viverrini, schistosoma mekongi and soil-transmitted helminthes on the Mekong islands, Southern Lao PDR. Infect Dis Poverty. 2017;6(1):131. [FREE Full text] [doi: 10.1186/s40249-017-0343-x] [Medline: 28866984]
21. Vaz Nery S, Qi J, Llewellyn S, Clarke NE, Traub R, Gray DJ, et al. Use of quantitative PCR to assess the efficacy of albendazole against necator americanus and ascaris spp. in Manufahi District, timor-leste. Parasit Vectors. 2018;11(1):373. [FREE Full text] [doi: 10.1186/s13071-018-2838-0] [Medline: 29954461]
22. Patel C, Coulibaly JT, Schulz JD, N'Gbesso Y, Hattendorf J, Keiser J. Efficacy and safety of ascending dosages of albendazole against in preschool-aged children, school-aged children and adults: a multi-cohort randomized controlled trial. EClinicalMedicine. 2020;22:100335. [FREE Full text] [doi: 10.1016/j.eclinm.2020.100335] [Medline: 32405623]
23. Foodborne trematode infections. WHO. 2022. URL: https://www.who.int/news-room/fact-sheets/detail/foodborne-trematode-infections [accessed 2023-10-11]
24. Sayasone S, Meister I, Andrews JR, Odermatt P, Vonghachack Y, Xayavong S, et al. Efficacy and safety of praziquantel against light infections of opisthorchis viverrini: a randomized parallel single-blind dose-ranging trial. Clin Infect Dis. 2017;64(4):451-458. [doi: 10.1093/cid/ciw785] [Medline: 28174906]
25. Lovis L, Mak T, Phongluxa K, Ayé Soukhathammavong P, Vonghachack Y, Keiser J, et al. Efficacy of praziquantel against schistosoma mekongi and opisthorchis viverrini: a randomized, single-blinded dose-comparison trial. PLoS Negl Trop Dis. 2012;6(7):e1726. [FREE Full text] [doi: 10.1371/journal.pntd.0001726] [Medline: 22848766]
26. Brigden A, Parslow RM, Linney C, Higson-Sweeney N, Read R, Loades M, et al. How are behavioural interventions delivered to children (5-11 years old): a systematic mapping review. BMJ Paediatr Open. 2019;3(1):e000543. [FREE Full text] [doi: 10.1136/bmjpo-2019-000543] [Medline: 31909219]
27. Nash R, Patterson K, Flittner A, Elmer S, Osborne R. School-based health literacy programs for children (2-16 years): an international review. J Sch Health. 2021;91(8):632-649. [doi: 10.1111/josh.13054] [Medline: 34096058]
28. Ayi I, Nonaka D, Adjovu JK, Hanafusa S, Jimba M, Bosompem KM, et al. School-based participatory health education for malaria control in Ghana: engaging children as health messengers. Malar J. 2010;9:98. [FREE Full text] [doi: 10.1186/1475-2875-9-98] [Medline: 20398416]
29. Health promoting schools. WHO. 2022. URL: https://www.who.int/health-topics/health-promoting-schools [accessed 2023-10-01]
30. Mationg MLS, Williams GM, Tallo VL, Olveda RM, Aung E, Alday P, et al. "The magic glasses philippines": a cluster randomised controlled trial of a health education package for the prevention of intestinal worm infections in school children. Lancet Reg Health West Pac. 2022;18:100312. [FREE Full text] [doi: 10.1016/j.lanwpc.2021.100312] [Medline: 35024651]
31. Bieri FA, Gray DJ, Williams GM, Raso G, Li Y, Yuan L, et al. Health-education package to prevent worm infections in Chinese school children. N Engl J Med. 2013;368(17):1603-1612. [doi: 10.1056/NEJMoa1204885] [Medline: 23614586]
32. Dwipayanti N, Phung T, Rutherford S, Chu C. Towards sustained sanitation services: a review of existing frameworks and an alternative framework combining ecological and sanitation life stage approaches. J Water Sanit Hyg Dev. 2017;7(1):25-42. [doi: 10.2166/washdev.2017.086]
33. Sekhon M, Cartwright M, Lawes-Wickwar S, McBain H, Ezra D, Newman S, et al. Does prospective acceptability of an intervention influence refusal to participate in a randomised controlled trial? An interview study. Contemp Clin Trials Commun. 2021;21:100698. [FREE Full text] [doi: 10.1016/j.conctc.2021.100698] [Medline: 33537506]
34. Heitzinger K, Hawes SE, Rocha CA, Alvarez C, Evans CA. Assessment of the feasibility and acceptability of using water pasteurization indicators to increase access to safe drinking water in the peruvian amazon. Am J Trop Med Hyg. 2020;103(1):455-464. [FREE Full text] [doi: 10.4269/ajtmh.18-0963] [Medline: 32372750]

XSL•FO
RenderX

35. Hussain F, Luby SP, Unicomb L, Leontsini E, Naushin T, Buckland AJ, et al. Assessment of the acceptability and feasibility of child potties for safe child feces disposal in rural Bangladesh. Am J Trop Med Hyg. 2017;97(2):469-476. [doi: 10.4269/ajtmh.15-0932] [Medline: 28722606]
36. Sultana F, Unicomb LE, Nizame FA, Dutta NC, Ram PK, Luby SP, et al. Acceptability and feasibility of sharing a soapy water system for handwashing in a low-income urban community in Dhaka, Bangladesh: a qualitative study. Am J Trop Med Hyg. 2018;99(2):502-512. [FREE Full text] [doi: 10.4269/ajtmh.17-0672] [Medline: 29893204]
37. Sekhon M, Cartwright M, Francis JJ. Acceptability of healthcare interventions: an overview of reviews and development of a theoretical framework. BMC Health Serv Res. 2017;17(1):88. [FREE Full text] [doi: 10.1186/s12913-017-2031-8] [Medline: 28126032]
38. Sekhon M, Cartwright M, Francis JJ. Development of a theory-informed questionnaire to assess the acceptability of healthcare interventions. BMC Health Serv Res. 2022;22(1):279. [FREE Full text] [doi: 10.1186/s12913-022-07577-3] [Medline: 35232455]
39. National Health and Medical Research Council, Australian Research Council and Universities Australia. National Statement on Ethical Conduct in Human Research. Canberra. National Health and Medical Research Council; 2023.
40. Ajzen I. The theory of planned behavior. Organ Behav Hum Decis Process. 1991;50(2):179-211. [doi: 10.1016/0749-5978(91)90020-t]
41. Schunk DH. Social cognitive theory. In: Harris KR, Graham S, Urdan T, McCormick CB, Sinatra GM, Sweller J, editors. APA Educational psychology Handbook, Vol 1 Theories, Constructs, and Critical Issues. Washington, DC. American Psychological Association; 2012:101-123.
42. Elo S, Kyngäs H. The qualitative content analysis process. J Adv Nurs. 2008;62(1):107-115. [doi: 10.1111/j.1365-2648.2007.04569.x] [Medline: 18352969]
43. Jensen A, Bonde LO. An arts on prescription programme: perspectives of the cultural institutions. Community Ment Health J. 2020;56(8):1473-1479. [doi: 10.1007/s10597-020-00591-x] [Medline: 32100154]
44. Varni JW, Limbers CA, Burwinkle TM. How young can children reliably and validly self-report their health-related quality of life?: an analysis of 8,591 children across age subgroups with the PedsQL 4.0 generic core scales. Health Qual Life Outcomes. Jan 03, 2007;5:1. [FREE Full text] [doi: 10.1186/1477-7525-5-1] [Medline: 17201920]
45. Riley AW. Evidence that school-age children can self-report on their health. Ambul Pediatr. 2004;4(4 Suppl):371-376. [doi: 10.1367/A03-178R.1] [Medline: 15264962]
46. Sturgess J, Rodger S, Ozanne A. A review of the use of self-report assessment with young children. British Journal of Occupational Therapy. 2016;65(3):108-116. [doi: 10.1177/030802260206500302]

Abbreviations

cRCT: cluster randomized controlled trial
FGD: focus group discussion
KAP: knowledge, attitudes, and practices
KII: key informant interviews
LMB: Lower Mekong Basin
MDA: mass drug administration
MGLM: Magic Glasses Lower Mekong
MGO: Magic Glasses Opisthorchiasis
OV: Opisthorchis viverrini
PDR: People’s Democratic Republic
SAC: school-aged children
STH: soil-transmitted helminths
TFA: Theoretical Framework of Acceptability
WASH: water, sanitation, and hygiene
WHO: World Health Organization

XSL•FO
RenderX

Edited by A Mavragani; submitted 11.12.23; peer-reviewed by F Sherine Ganem Dos Santos; comments to author 07.03.24; revised version received 27.03.24; accepted 17.04.24; published 16.09.24

Please cite as:
O'Connor SY, Mationg ML, Kelly MJ, Williams GM, Clements ACA, Sripa B, Sayasone S, Khieu V, Wangdi K, Stewart DE, Tangkawattana S, Suwannatrai AT, Savathdy V, Khieu V, Odermatt P, Gordon CA, Wannachart S, McManus DP, Gray DJ
Examining the Acceptability of Helminth Education Packages "Magic Glasses Lower Mekong" and "Magic Glasses Opisthorchiasis" and Their Impact on Knowledge, Attitudes, and Practices Among Schoolchildren in the Lower Mekong Basin: Protocol for a Cluster Randomized Controlled Trial
JMIR Res Protoc 2024;13:e55290
URL: https://www.researchprotocols.org/2024/1/e55290
doi: 10.2196/55290
PMID:

©Suji Y O'Connor, Mary Lorraine Mationg, Matthew J Kelly, Gail M Williams, Archie CA Clements, Banchob Sripa, Somphou Sayasone, Virak Khieu, Kinley Wangdi, Donald E Stewart, Sirikachorn Tangkawattana, Apiporn T Suwannatrai, Vanthanom Savathdy, Visal Khieu, Peter Odermatt, Catherine A Gordon, Sangduan Wannachart, Donald P McManus, Darren J Gray. Originally published in JMIR Research Protocols (https://www.researchprotocols.org), 16.09.2024. This is an open-access article distributed under the terms of the Creative Commons Attribution License (https://creativecommons.org/licenses/by/4.0/), which permits unrestricted use, distribution, and reproduction in any medium, provided the original work, first published in JMIR Research Protocols, is properly cited. The complete bibliographic information, a link to the original publication on https://www.researchprotocols.org, as well as this copyright and license information must be included.

XSL•FO
RenderX
