# Supplementary material for: The Impact of “The Magic Glasses Opisthorchiasis” on Schoolchildren’s Knowledge, Attitudes and Practices Surrounding Opisthorchis viverrini in the Lower Mekong Basin, a Cluster-Randomised Controlled Trial
Source: Trop Med Infect Dis. 2026 Jun 24;11(7):174. doi: 10.3390/tropicalmed11070174 (PMC13417138; doi:10.3390/tropicalmed11070174)
Supplement: Supplementary file 1 [file tropicalmed-11-00174-s001.zip › Supp 2. OV KAP questionnaire scoring.pdf]

Supplementary Material S2. OV KAP questionnaire scoring "Magic Glasses Opisthorchiasis" cluster-randomized controlled trial.

This Supplementary Material was adapted from O'Connor SY, Mationg ML, Kelly MJ, Williams GM, Clements ACA, Sripa B et al. Examining the Acceptability of Helminth Education Packages "Magic Glasses Lower Mekong" and "Magic Glasses Opisthorchiasis" and Their Impact on Knowledge, Attitudes, and Practices Among Schoolchildren in the Lower Mekong Basin: Protocol for a Cluster Randomized Controlled Trial. JMIR Research Protocols. 2024 Sept 16;13:e55290. doi: 10.2196/55290; copyright © Suji Y O'Connor, Mary Lorraine Mationg, Matthew L Kelly, Gail M Williams, Archie CA Clements, Banchob Sripa, Somphou Sayasone, Virak Khieu, Kinley Wangdi, Donald E Stewart, Sirikachorn Tangkawattana Apiporn T Suwannatrai, Vanthanom Savathdy, Visal Khieu, Peter Odermatt, Catherine A Gordon, Sangduan Wannachart, Donald P McManus, Darren J Gray. Originally published in JMIR Research Protocols (http://www.researchprotocols.org), 16.09.2024. This was published and can be reproduced under the terms of Creative Commons Attribution (https://creativecommons.org/licenses/ by/4.0/).

**A. SURVEY IDENTIFICATION**<br>**To be filled in by research team:**		**Date: (yyyy–mm–dd) |__|__ |__|__| / |__|__| / |__|__|**
1	Village (Name and Code)	|__|__|
2	School (Name and Code)	|__|__|
3	Grade	
4	Class/Section	|__|__|
5	Student Number	|__|__|
**B. PERSONAL INFORMATION**		
1	First Name	
2	Last Name	
3	Sex	☐ 1 – Male ☐ 2 – Female
4	Date of Birth (yyyy–mm–dd)<br>If you can't remember, use 6 for the month/15 for the day of the month	|__|__ |__|__| / |__|__| / |__|__|
**C. KNOWLEDGE ABOUT LIVER FLUKE**<br>**Please let us know what you have heard and what you know about liver fluke.**		
1	Have you ever heard about the liver fluke or Opisthorchiasis?<br>(maximum score = 1)	☐ 0= No (Go to E1)<br>☐ 1= Yes **(1 point)**<br>☐ 2= Don't know (Go to E1)
2	If yes, where did you hear about liver fluke?	☐ 1 = Friend<br>☐ 2 = Poster

		☐ 3 = TV<br>☐ 4 = Radio<br>☐ 5 = Book<br>☐ 6 = Brochure<br>☐ 7 = School<br>☐ 8 = Nurse/Doctor<br>☐ 9 = Internet/social media<br>☐ 10 = Parents/Family<br>☐ 11 = Others, please specify _________________<br>☐ 12 = Unknown/Can't remember
3	Have you ever had liver fluke?	☐ 0= No<br>☐ 1= Yes<br>☐ 2= Don't know
4	Do you know somebody who had liver fluke?	☐ 0= No<br>☐ 1= Yes<br>☐ 2= Don't know
**D. TRANSMISSION, SYMPTOMS AND TREATMENT OF LIVER FLUKES (maximum score = 15)**		
1	How can you get liver fluke?<br>(maximum score = 1)	☐ 1= Mosquito bite<br>☐ 2= Swimming in the river/canal<br>☐ 3= Fishing<br>☐ 4= Playing with soil<br>☐ 5= Dirty hands<br>☐ 6= Eating raw/undercooked and fermented fish **(1 point)**<br>☐ 7= Others, specify<br>☐ 8= Don't know
2	Do you think liver fluke can make you sick?<br>(maximum score = 1)	☐ 0= No<br>☐ 1= Yes **(1 point)**<br>☐ 2= Don't know
3	What happens if you have liver fluke?<br>(maximum score = 3)	☐ 1= Liver fluke cancer **(1 point)**<br>☐ 2= Blindness<br>☐ 3= Fever **(1 point)**<br>☐ 4= High blood pressure<br>☐ 5= Feeling tired **(1 point)**<br>☐ 6= Slow growth<br>☐ 7= Others, specify:<br>☐ 8= Don't know
4	How can you prevent/avoid liver fluke?<br>(maximum score = 3)	☐ 0= Don't know<br>☐ 1= Using latrine **(1 point)**<br>☐ 2= Sleeping under a mosquito net<br>☐ 3= Doing exercise<br>☐ 4= Better sewerage system **(1 point)**<br>☐ 5= Avoid consumption of raw/undercooked fish **(1 point)**<br>☐ 6= Others, specify:
5	Do you think liver fluke can be treated?<br>(maximum score = 1)	☐ 0= No<br>☐ 1= Yes **(1 point)**

		☐ 2= Don't know
6	If yes, where can you go for treatment<br>(maximum score = 3)	☐ 0= Don't know<br>☐ 1= School **(1 point)**<br>☐ 2= Health Center **(1 point)**<br>☐ 3= Hospital **(1 point)**<br>☐ 4= Traditional or Faith Healer
7	Can taking medicine cure liver flukes forever?<br>(maximum score = 1)	☐ 0 = No **(1 point)**<br>☐ 1 = Yes<br>☐ 2 = Don't know
7	What do you think is the treatment for the liver fluke?<br>(maximum score = 1)	☐ 0= Don't know<br>☐ 1= Aspirin<br>☐ 2= Praziquantel **(1 point)**<br>☐ 3= Albendazole<br>☐ 4= Others, specify:
**E. ATTITUDE ABOUT LIVER FLUKE (maximum score = 7)**		
1	Do you believe that you are likely to be infected with liver fluke?<br>(maximum score = 1)	☐ 0= No<br>☐ 1= Yes **(1 point)**<br>☐ 2= Don't know
2	If yes, what is your risk or chance of getting liver fluke?<br>(maximum score = 1)	☐ 0= None (0 point)<br>☐ 1= Low Possibility (0 point)<br>☐ 2= Medium Possibility **(0.5 point)**<br>☐ 3= High Possibility **(1 point)**
3	Would you be worried if you get infected with liver fluke?<br>(maximum score = 1)	☐ 0= No<br>☐ 1= Yes **(1 point)**<br>☐ 2= Don't know
4	What do you think is the chance that your neighbour will get infected with liver fluke?<br>(maximum score = 1)	☐ 0= None<br>☐ 1= Low<br>☐ 2= Medium **(0.5 point)**<br>☐ 3= High **(1 point)**<br>☐ 4= Don't know
5	How bad do you think liver fluke is as a disease in your village?<br>(maximum score = 1)	☐ 0= Not severe<br>☐ 1= Low severity<br>☐ 2= Medium severity **(0.5 point)**<br>☐ 3= High severity **(1 point)**<br>☐ 4= Don't know
6	Liver fluke is a problem in your village<br>(maximum score = 1)	☐ 0= Strongly disagree<br>☐ 1=Disagree<br>☐ 2= Agree **(0.5 point)**<br>☐ 3= Strongly Agree **(1 point)**
7	Have you ever been treated for liver flukes?<br>(maximum score = 1)	☐ 0= No<br>☐ 1= Yes **(1 point)**<br>☐ 2= Don't know
7.1	If yes, how many times?	☐ 1= Once<br>☐ 2= Twice<br>☐ 3= Three times

		☐ 4= More than 3 times (specify:________)<br>☐ 5= Don't know
7.2	If yes, where did you go for treatment? (multiple answers possible)	☐ 1= Health Center<br>☐ 2= Hospital<br>☐ 3= Traditional or Faith Healer<br>☐ 4= Local grocery<br>☐ 5= Others, specify: ____________________
8	When was your last treatment with PZQ?	☐ 1=Less than 1 month<br>☐ 2=1-6 months<br>☐ 3=7-12 months<br>☐ 4=> 1 year
**F. HEALTH EDUCATION RELATED TO LIVER FLUKE**		
1	Has your teacher already told you about liver flukes?	☐ 0= No<br>☐ 1= Yes<br>☐ 2= Don't know
2	Have you watched video (in social media, Youtube) on liver flukes?	☐ 0= No<br>☐ 1= Yes<br>☐ 2= Don't know
3	Have you ever done an assignment on liver flukes?	☐ 0= No<br>☐ 1= Yes<br>☐ 2= Don't know
4	Have you told your parents, sisters and brothers about liver flukes?	☐ 0= No<br>☐ 1= Yes<br>☐ 2= Don't know
5	Have your parents told you about the liver flukes?	☐ 0= No<br>☐ 1= Yes<br>☐ 2= Don't know
**G. RISK–TAKING BEHAVIOUR ABOUT LIVER FLUKE (maximum score = 8)**		
1	Do you eat raw or live fish?<br>(maximum score = 1)	☐ 0= No<br>☐ 1= Yes **(1 point)**<br>☐ 2= Don't know
2	If yes, how often do you consume raw fish? (for example, koi pla or other raw fish dishes)<br>(maximum score = 2.5)	☐ 1= less than once per month **(0.5 point)**<br>☐ 2= 1-3 per month **(1 point)**<br>☐ 3= 1-3 per week **(1.5 points)**<br>☐ 4= 4-6 per week **(2 points)**<br>☐ 5= Every day or ≥ 1 per day **(2.5 points)**
3	Does your family consume raw undercooked/fermented fish?<br>(maximum score = 1)	☐ 0= No<br>☐ 1= Yes **(1 point)**<br>☐ 2= Don't know
4	How often does your family prepare or consume raw fish?<br>(maximum score = 2.5)	☐ 1= Less than once per month (0.5 point)<br>☐ 2= 1–3 per month **(1 point)**<br>☐ 3= 1–3 per week **(1.5 points)**<br>☐ 4= 4–6 per week **(2 points)**<br>☐ 5= Every day or ≥ 1 per day **(2.5 points)**

5	How does your family dispose of your waste food?<br>(maximum score = 1)*	☐ 1= Disposal by giving it to dogs or cats **(1 point)**<br>☐ 2= Discarding to an open pit to which animals had access **(1 point)**<br>☐ 3= Covered compost pit at home<br>☐ 4= Throw in waste bin<br>☐ 5= Throw anywhere **(1 point)**<br>☐ 6= Others, specify

*This question could score a maximum of 1 point. In cases where one or multiple unsafe options were ticked, participants would receive a score of 1. In cases where both safe and unsafe options were ticked, the safe responses would negate the unsafe responses. For example, if a participant ticked response 1 (disposal by giving to dogs or cats) and response option 4 (waste bin), then they would score 0. If a participant ticked response 1 (disposal by giving to dogs or cats), response option 2 (discarding to an open pit), and response option 4 (waste bin), then they would score 1.
